# Intelligent medicine in focus: the 5 stages of evolution in robot-assisted surgery for prostate cancer in the past 20 years and future implications

**DOI:** 10.1186/s40779-024-00566-z

**Published:** 2024-08-21

**Authors:** Jia-Kun Li, Tong Tang, Hui Zong, Er-Man Wu, Jing Zhao, Rong-Rong Wu, Xiao-Nan Zheng, Heng Zhang, Yi-Fan Li, Xiang-Hong Zhou, Chi-Chen Zhang, Zi-Long Zhang, Yi-Hang Zhang, Wei-Zhe Feng, Yi Zhou, Jiao Wang, Qi-Yu Zhu, Qi Deng, Jia-Ming Zheng, Lu Yang, Qiang Wei, Bai-Rong Shen

**Affiliations:** 1 Department of Urology, West China Hospital, Sichuan University, Chengdu, 610041 China; 2 Institutes for Systems Genetics, Frontiers Science Center for Disease-Related Molecular Network, West China Hospital, Sichuan University, Chengdu, 610041 China; 3 Department of Computer Science and Information Technologies, Elviña Campus, University of A Coruña, 15001 A Coruña, Spain; 4 Chinese Academy of Medical Science Oxford Institute, Nuffield Department of Medicine, University of Oxford, Oxford, OX1 2JD UK; 5 Department of Radiology, National Clinical Research Center for Geriatric Diseases/the Second Medical Center of Chinese PLA General Hospital, Beijing, 100853 China

**Keywords:** Robotic surgical system, Prostate cancer, Robot-assisted radical prostatectomy, Intelligent medicine

## Abstract

Robot-assisted surgery has evolved into a crucial treatment for prostate cancer (PCa). However, from its appearance to today, brain-computer interface, virtual reality, and metaverse have revolutionized the field of robot-assisted surgery for PCa, presenting both opportunities and challenges. Especially in the context of contemporary big data and precision medicine, facing the heterogeneity of PCa and the complexity of clinical problems, it still needs to be continuously upgraded and improved. Keeping this in mind, this article summarized the 5 stages of the historical development of robot-assisted surgery for PCa, encompassing the stages of emergence, promotion, development, maturity, and intelligence. Initially, safety concerns were paramount, but subsequent research and engineering advancements have focused on enhancing device efficacy, surgical technology, and achieving precise multi modal treatment. The dominance of da Vinci robot-assisted surgical system has seen this evolution intimately tied to its successive versions. In the future, robot-assisted surgery for PCa will move towards intelligence, promising improved patient outcomes and personalized therapy, alongside formidable challenges. To guide future development, we propose 10 significant prospects spanning clinical, research, engineering, materials, social, and economic domains, envisioning a future era of artificial intelligence in the surgical treatment of PCa.

**Supplementary Information:**

The online version contains supplementary material available at 10.1186/s40779-024-00566-z.

## Background

Prostate cancer (PCa) is one of the predominant malignant tumors in men and the top of 5 leading causes of death worldwide [1]. For localized and regional stage PCa, the 5-year survival rate after treatment is greater than 99%. However, once PCa progresses to the distant metastasis stage, the 5-year survival rate drops to only 32% [2]. Thus, timely intervention and treatment in the early stages of the disease are essential for patients’ safety.

The mainstream treatment for localized PCa is robot-assisted radical prostatectomy (RARP), which greatly improves and ensures patient survival while effectively treating early-stage PCa [3]. Since the Food and Drug Administration (FDA) approved the da Vinci robot-assisted surgical system in 2000, it has dominated the market for over 20 years. Despite new robotic systems emerging, the da Vinci system remains synonymous with robot-assisted surgery. Over two decades, technological advancements and instrument updates have driven medical evolution.

In recent years, there has been a growing interest in the medical field regarding artificial intelligence (AI) technologies, specifically related to brain-computer interfaces [4], virtual reality [5], 5G technology [6], and the metaverse [7].

However, treating PCa faces challenges due to tumor differences and complex clinical problems, limiting decision-making. In the context of big data, AI, and interdisciplinary development, we reviewed the evolution of robot-assisted surgery for PCa, summarizing its 5 stages. We suggest that intelligent medicine is likely the future path for robot-assisted PCa surgery.

## Characteristics and applications of robotic surgical system

In 2001, the first reported robot-assisted laparoscopic radical prostatectomy marked the rapid development of this important alternative for the surgery of PCa in many countries [8]. The characteristics of each generation of surgical instruments are essential to this development (Additional file 1: Fig. S1, Table S1).

It possesses a superb three-dimensional (3D) visual system. Compared to traditional open surgery, the magnification and 3D high-definition visual system under the robotic surgical system’s view provide a clearer surgical view. This helps doctors to perform delicate operations more effectively.

The robotic surgical system has excellent flexibility, with a range of motion more extensive than the human wrist. The da Vinci surgical system has a wrist range of 360°. It can perform precise surgical operations such as vascular anastomosis and nerve protection in radical prostatectomy. Robotic surgical system has also improved ergonomics by eliminating hand tremors, making fine surgical procedures more smooth/efficient. This precision is a hallmark of the robotic surgical system.

The robotic surgical system also has been assisted by an excellent overall ecosystem, such as intraoperative ultrasound imaging, 3D elastic augmented reality or real-time augmented reality, and other comprehensive auxiliary tools, which have promoted the development of RARP [9]. As technology continues to develop, the robot surgical ecosystem will become more comprehensive and achieve more functions.

Additionally, the robotic surgical system is also representative of minimally invasive surgery. With continuous advancements in technology, the da Vinci surgical system has created a single-port (SP) robot platform. SP-RARP had similarly high safety compared to multi-port (MP) surgery and could reduce patients’ hospitalization times [10]. Moreover, it is less invasive with fewer incisions, significantly reducing patient complications.

Although the robotic surgical system has excellent advantages, there are also limitations. First, its cost is relatively high compared to traditional surgery. Second, it has certain requirements for the operator. The emergence of technology represents a learning curve change. Third, it requires a well-coordinated team to support the medical institution. Moreover, the robotic equipment may experience mechanical failure or systemic errors, resulting in adverse reactions. Additionally, patients need to maintain a special posture during surgery and use greenhouse gases, which may cause complications, such as eye- or neural-related problems, but these do not differ significantly from traditional surgery.

In summary, under objective conditions, robotic surgical system exhibits meticulousness, flexibility, softness, and controllability, and can be used for a variety of surgical operations.

## Five stages of development of robot-assisted surgery for PCa

### Stage I: emergence period, security evidence-based paradigm

Since 1996, the da Vinci robot-assisted surgical system has been developed in the United States (US) and officially approved by the US FDA for use in radical prostatectomy in 2001 [8]. Therefore, over 90% of the researches and publications in this stage came from the US.

Research primarily focused on validating the safety of this technology and evaluating surgeons’ proficiency with this surgical approach. Therefore, this stage of research focused on the details of the surgical procedures and the learning curve to evaluate both the safety and advantages of RARP. As the robotic surgical system began to show its advantages, the prognosis for PCa patients improved. For example, a study of 143 patients with clinically localized PCa who underwent RARP found that it offers early recovery and oncologic outcomes comparable to open surgery, with benefits such as shorter hospital stays and high continence rates within a year [11]. In addition, patients who underwent RARP were more satisfied than those who underwent endoscopic surgery [odd ratio (*OR*) = 3.02, 95% confidence interval (CI) 1.50–6.07] [12]. One study has shown that RARP might result in better postoperative outcomes, particularly in terms of urinary incontinence and sexual dysfunction when compared to traditional surgery [13].

**Fig. 1:**
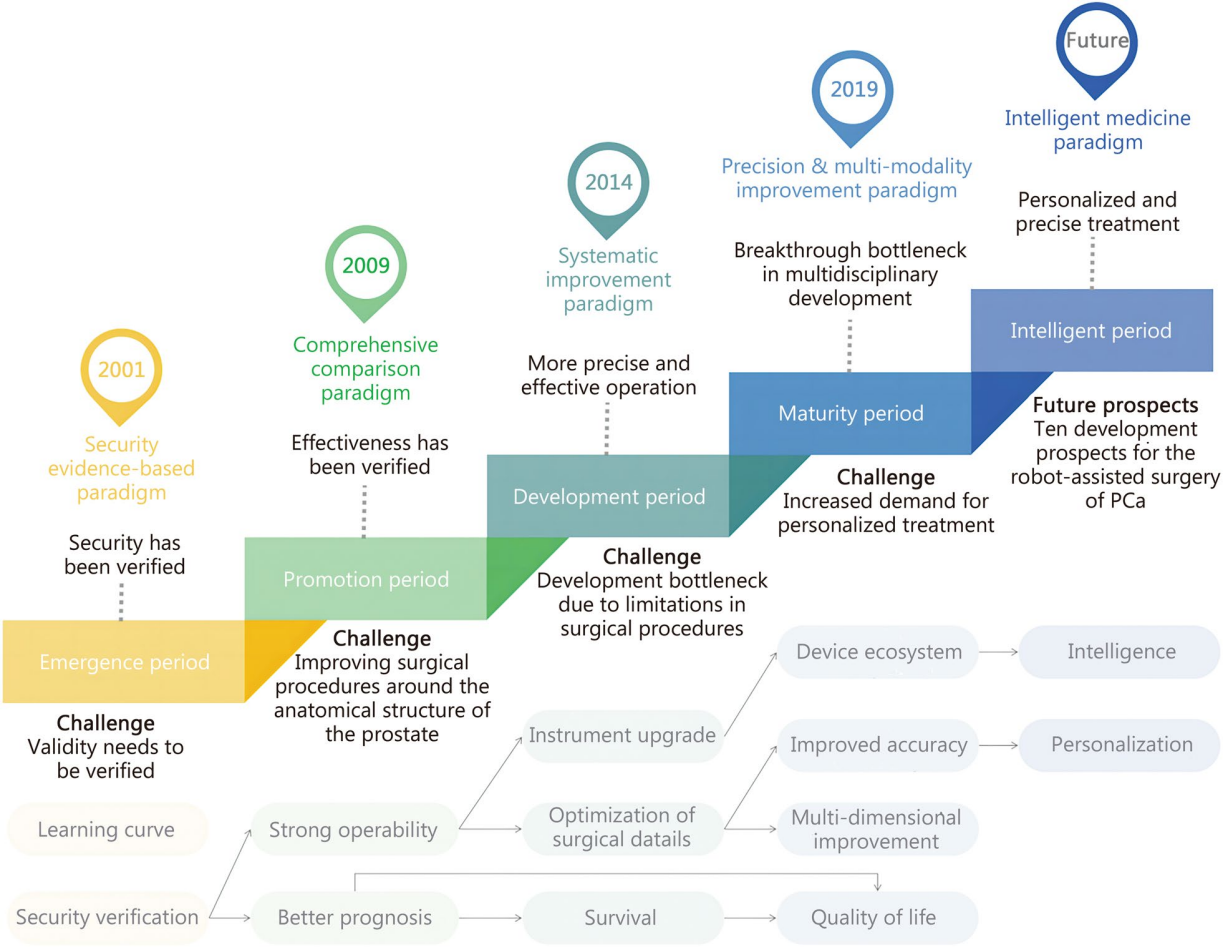
The characteristics and key points of the 5 stages of the development of robot-assisted surgery. Under the corresponding research and development paradigm, research at each stage has made breakthroughs and faced new challenges. The emergence period, which began in 2001, focused on the evidence-based security of robotic surgical systems. Since entering the promotion period in 2009, due to the recognition of safety in the conclusions drawn from the previous stage of research, this stage focused on a comprehensive comparison between robot-assisted surgery and traditional surgery. Robotic surgical systems had strong operability and better patient prognosis. Since 2014, it entered the development period. Robot-assisted surgery has entered a comprehensive improvement. Based on strong operability, on the one hand, equipment upgrade researches were carried out, and on the other hand, relying on good equipment, fine surgical operations were expanded. A better prognosis was reflected in survival rate and quality of life. Since 2019, it entered the maturity period. The control of surgical operation details made treatment more precise, and the improvement of equipment made the patient’s diagnosis and treatment process more multi-dimensional and multi-modal. Finally, in the future, the development of robot-assisted surgery will enter the era of intelligence, and intelligent medicine will make patient diagnosis and treatment more personalized. PCa prostate cancer

At this stage, the focus is on the safety and utility of robot-assisted surgery. Evidence shows that RARP reduces blood loss, pain, and hospital stays while allowing early activity [14]. However, research is still needed to verify if it achieves treatment effects comparable to traditional surgery, which is crucial for its further development. Overall, RARP has made breakthrough progress from scratch in nearly 10 years on that time. With the application of clinical trials in multiple centers, research has found that it seems to have good safety and advantages [15]. Thus, it has been steadily introduced into clinical practice (Fig. 1**, **Table 1).

**Table 1:** The 5 stages of evolution and the future in the context of intelligent medicine of robot-assisted surgery for PCa

Items	Stage 1: emergence period	Stage 2: promotion period	Stage 3: development period	Stage 4: maturity period	Stage 5 (future): intelligent period
Important event	Da Vinci approved	Da Vinci Si	Da Vinci Xi	Promotion of Da Vinci SP	Artificial intelligence (AI)-intelligent medicine
Point of time	2001	2009	2014	2019	Future
Research core	Security and learning curve	Relative advantage value	Surgical techniques and procedures	Surgical ecology	Personalization and precision intelligence
Research objective	Addressing security	System credibility	Improve operability	Improve accuracy	Future intelligence
Equipment characteristics	Mobility, multiple images	Dual console, analog controller, and intraoperative fluorescence	Flexibility, accuracy, and imaging clarity	Multi-port to single port. For abdominal surgery with narrow range	Lower price, multiple device options, and more widespread application
Characteristics of surgical development	Focusing on robotic surgery to verify whether the prognosis reaches the level of traditional surgery	Robot surgery has a better therapeutic effect than traditional surgery	Targeting more operational skills and details	Multi-dimensional and multimodal applications, with more details	Combining with AI and application technology
Clinical orientation	Surgical safety and complications	Survival and biochemical recurrence	Quality of life	Multi-dimensional prognosis	Personalized treatment plan
Equipment	Da Vinci	Da Vinci	Da Vinci	Da Vinci and other robotic surgical systems	Multiple robot surgical systems
Research paradigm	Security evidence-based paradigm	Comprehensive comparison paradigm	Systematic improvement paradigm	Precision & multi-modality improvement paradigm	Intelligent medicine paradigm

### Stage II: promotional period, comprehensive comparison paradigm

During this stage, there was a noticeable increase in research quantities. In 2009, the third-generation da Vinci Si surgical system was introduced, improving the learning curve, controllability, and safety of surgeries [16]. This enhancement boosted the effectiveness of RARP. With the promotion of this technology, more countries and regions began to adopt RARP.

Research during this period addressed the operational challenges of PCa surgery and improved patient prognosis. Robot-assisted surgery was promoted globally, with multi-center comparisons to traditional surgeries to validate its superiority. The robotic system’s flexibility and precision led to refined operations, focusing on lymph node and nerve preservation. Research expanded to include patient survival and postoperative prognosis indicators, such as biochemical recurrence (BCR) and positive surgical margins (PSM). Additionally, study on the quality of life after robot-assisted surgery showed that RARP could provide better tumor and functional outcomes compared with traditional methods [17]. Research showed that the 12 months’ continence of robotic surgery was 1.35 times more than traditional surgery [18].

Moreover, some economic studies showed that while robot-assisted surgery has a shorter operation time, its cost is about 1.39 times of that of open surgery [19]. Thus, discussions about the efficiency of robot-assisted surgery considered its strengths and weaknesses, such as lower PSM, longer surgical time, higher costs, and more optimized operating room flow and economic solutions.

During this stage, comprehensive comparisons mostly relied on statistical models due to the different patient sets involved in various surgical methods [20]. Regression and correlation analyses supported numerous predictive studies, which laid the groundwork for AI application in robotic PCa surgery. While AI is superficially used in other medical models, it has significant potential in robotic surgery [21].

After validation, the robotic surgical system was promoted globally. Comparison studies highlighted its advantages, especially in long-term prognosis. However, RARP must continue to evolve beyond being superior to traditional surgery [22]. Despite ensuring patient survival, quality of life remains an issue. Improving the precision of anatomical structure processing in surgery is a current challenge.

### Stage III: development period, systematic improvement paradigm

Research in this phase experienced significant growth. The fourth-generation da Vinci Xi surgical system, introduced in 2014, initially had issues like lack of tactile feedback and long docking times. The latest da Vinci Xi system fixed these with a laser positioning system and non-colliding mechanical arms, making it suitable for complex operations and improving outcomes for PCa patients. As medical standards improved, the focus shifted from just survival to functional outcomes in treating localized PCa. Consequently, research began to prioritize quality of life over BCR. Efforts were made to refine surgical details because intraoperative procedures directly affect postoperative prognosis and quality of life [23]. These details included Retzius-sparing RARP, which effectively reduced BCR [24], surgical procedures like bladder neck protection, fascial reconstruction, and pubovesical complex reconstruction to improve postoperative urinary incontinence, and techniques for preserving nerves and blood vessels to enhance postoperative function. Lymph node dissection might not be suitable for low-risk PCa patients due to mostly negative findings, but it may benefit intermediate to high-risk PCa patients. Additionally, some studies explored other intraoperative details, such as patient positioning, to reduce ocular and neurological complications and improve prognosis [25, 26].

To improve patients’ postoperative quality of life, research focused on protecting nerves, blood vessels, and urethral structures during surgery to enhance outcomes (Fig. 1, Table 1). However, the surgical treatment of PCa appeared to reach saturation, and robotic surgery faced new development challenges. At this time, AI began to emerge in the medical field, such as for medical information system detection and prediction, offering new possibilities for intelligent PCa robot surgery.

### Stage IV: maturity period, precision & multi-modality improvement paradigm

Due to the previous bottleneck, robot surgery for PCa has started to seek breakthroughs beyond the surgery itself. The 2018 approval of the da Vinci SP system, followed by the release of the cohort in 2019, marked the beginning of a new era in RARP [27]. This phase has seen multi-dimensional and multi-modality developments in surgery. Advances in engineering and assistive technologies have expanded surgical research. For example, ^18^F-DCFPyL positron emission tomography (PET)/computed tomography (CT) accurately diagnoses and predicts postoperative outcomes, ensuring precise treatment [28]. Multi-parametric magnetic resonance imaging predicts tumor prognosis and postoperative urinary incontinence. Intraoperative frozen section technology with fluorescent confocal microscopy improves margin prediction and supports 3D-guided surgery. Augmented reality-3D uses augmented reality for personalized nerve-sparing. β-rays and indocyanine green guide precise localization, and low-intensity extracorporeal shock waves aid rehabilitation. Advances in intrapelvic structure analysis have improved urinary control and balanced oncological and sexual outcomes. Robotic surgical systems have also seen breakthroughs. While the da Vinci system is the mainstream, its failure rates range from 0.4% to 3.7% [29]. More systems are being introduced into clinical practice. The Hugo™ robot-assisted surgery system is safe, reliable, and efficient, with acceptable perioperative outcomes and early urinary incontinence recovery. The KangDuo surgical robot-01 (KD-SR-01) system is also feasible, safe, and effective for localized PCa [30]. A study showed that although KangDuo RARP had longer operation times, it achieved similar short-term outcomes compared to da Vinci Si [31]. The Hinotori™ surgical robot system has also demonstrated safety. Additionally, robotic systems have established remote surgical platforms for future clinical use. These systems promote balanced development and competition, reducing costs and bringing more benefits and precision to future RARP, supplementing the robot-assisted surgery library (Fig. 1, Table 1).

Intelligent medical applications have been applied and developed in the treatment of PCa. Research paradigms now include data-driven methods and deep learning. With big data support, AI methods like deep learning are increasingly used in PCa research. For example, AI with neural networks diagnoses and evaluates tumors in RARP patients [32]. Machine learning algorithms predict postoperative outcomes more reliably than traditional models, or use AI to identify patient characteristics for functional outcome predictions. There are also studies on AI application in PCa patient management, indicating robot-assisted surgery is entering the AI era [33, 34].

In summary, robot-assisted surgery for PCa has matured. Multidisciplinary cooperation has led to multimodal development. Engineering technology advances have enriched the robotic surgery ecosystem, making clinical practice more precise and multi-dimensional. These advances have elevated the surgical treatment of PCa. The demand for intelligent, personalized, and precise treatment is growing.

### Stage V: intelligent period, intelligent medicine paradigm

Research on RARP is expected to continue increasing as the incidence of PCa continues to rise. From the estimated new cases of 288,300 in the US by 2023 [1], it is likely that the number of patients is increasing due to changes in population structure [35]. Additionally, the use of robot-assisted surgery is expected to become more widespread worldwide due to economic and scientific progress, leading to a wider coverage of precision medicine, which will promote the wider application of robot-assisted surgical systems.

Most importantly, as the application of AI in the medical field becomes more widespread and the model of medicine + AI matures, AI is expected to rise in urological surgery.

The integration of surgical robots with AI technology holds vast potential, as evidenced by its multifaceted application in various areas such as brain-computer interfaces [4], virtual reality [5], 5G technology [6], and the emerging metaverse [7]. Effectively harnessing this technology improves surgical precision, safety, efficiency, and outcomes, enhancing patient experience and driving healthcare innovation. Integration requires extensive research, testing, and standardized protocols for safety and reliability. Addressing these ensures effective use of surgical robots and AI, advancing patient care and medical science. Future RARP applications include as below.

First, medical decision-making: AI assists in making more scientific and accurate decisions, overcoming clinical experience limitations, and offering precise, personalized treatment [36].

Second, at the technical level: once the decision is made to use RARP, the robotic surgical system with its hardware and software system dimensions and supporting facilities ecosystem are involved [37]. AI can be integrated with software systems and supporting facilities to provide intelligent guidance for surgeries. This can lead to better postoperative survival and functional benefits for patients, and make RARP easier to learn for beginners. Future robot-assisted surgical devices will likely become more intelligent, continuously improving surgical accuracy and efficiency through machine learning, deep learning, and human operation simulations [38].

Third, at the research level: using existing research paradigms, the overall trend will be combined with AI methods, such as intelligent knowledge platforms [39]. On one hand, as data accumulates, AI methods can be applied to big data calculations and scientific and reasonable conclusions can be drawn. On the other hand, research will extend to multiple dimensions, and existing research can be used as knowledge to guide algorithm innovation [40]. In the future, RARP will also involve the integration of multiple disciplines, such as robotics technology, biomedical engineering, image processing, and human–computer interaction. It will enter into an era of multi-disciplinary cross-over, data-driven, knowledge-guided AI (Fig. 1, Table 1).

## Discussion and future prospects

In the evolution of robot-assisted surgery for PCa, there has been a progression from the emergence of robotic surgical devices to their widespread global use as the primary approach for PCa treatment [3].

The operating room lacks a professional team with limited personnel in many cases [41]. Although the robotic surgical equipment’s technological components are constantly evolving, surgeons need to have excellent and adaptive skills for surgical treatment [42]. Therefore, medical institutions should mandate requirements for robotic surgery teams, limiting participation to experienced and competent members. They should update personnel requirements to match evolving robotic surgical equipment and skills. Medical and educational institutions must train surgical teams to improve their proficiency with robotic PCa surgery. The skills and roles of team members are crucial and must not be overlooked, even with AI’s advancements in clinical practice [43, 44].

In addition, although robot surgery is generally more expensive than laparoscopic surgery in terms of cost and patient expenses, this issue needs to be viewed dialectically [5]. On the one hand, patients with PCa may achieve better prognosis through robotic surgery and be able to return to work earlier to make up for some of the surgical expense [46]. Patients recover faster and return to work sooner, potentially balancing hospitalization costs. On the other hand, hospitals can promote this technology to improve efficiency. Despite high equipment costs, it offers significant medical benefits and high turnover efficiency. More interdisciplinary research in sociology and economics on PCa robotic surgery is needed to improve clinical efficiency.

**Fig. 2:**
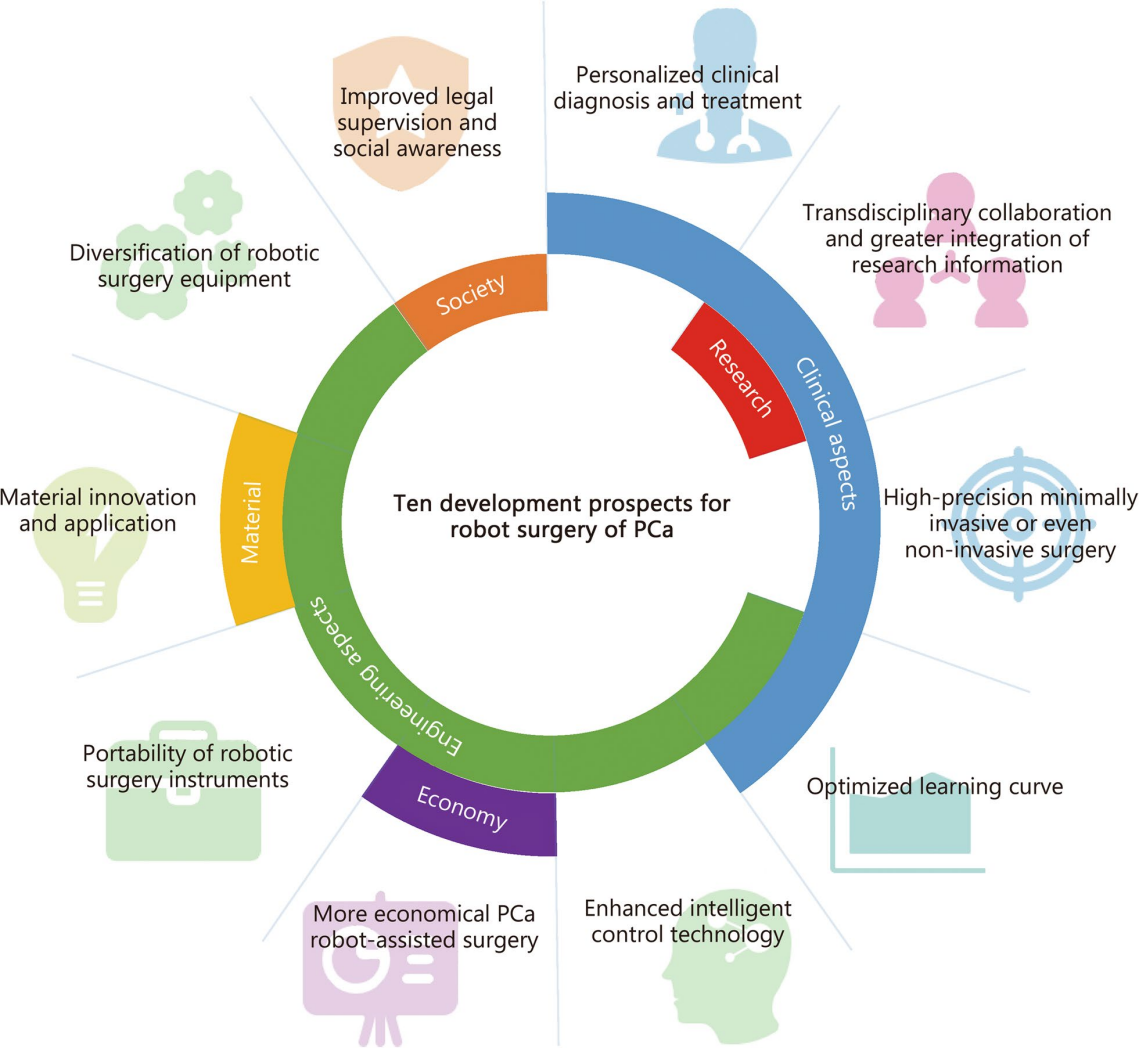
Ten development prospects for robot surgery of PCa. The figure showed 10 aspects of the future development of robot surgery, which intersected different fields. The names of different fields are displayed on the center circle. PCa prostate cancer

However, future challenges for RARP include clinical, engineering, material, and social aspects. Clinical decisions affect patient outcomes, including the need for robot-assisted surgery and avoiding over-treatment. Engineering challenges like safety, cost-effectiveness, and flexibility are crucial. Societal acceptance and legal regulations are also important for RARP’s future development. These challenges aim to improve healthcare quality. Given the current era, we have summarized 10 development prospects for the robot-assisted surgery of PCa (Fig. 2).

1. Personalizing diagnosis and treatment for PCa due to its heterogeneity and patient condition complexity.
2. Advancing minimally invasive surgery with robotic systems, reducing incisions and tissue damage.
3. Enhancing AI for real-time monitoring and better outcomes.
4. Optimizing learning curves with simplified, standardized procedures, and machine learning.
5. Making robotic instruments more portable and compact.
6. Increasing diversity of robotic surgical equipment from more manufacturers.
7. Reducing costs of PCa robotic surgery through technological advancements and competition.
8. Innovating materials to improve effectiveness and reliability of robotic surgery.
9. Encouraging transdisciplinary collaboration and integration of research.
10. Strengthening legal oversight and social awareness for robotic surgery applications.

## Conclusions

Robot-assisted surgery is now essential for PCa treatment. RARP has evolved through 4 stages: safety, equipment value, surgical technique, and precise treatment with multi-modality development. In the future, RARP is expected to enter the AI age. Addressing clinical, engineering, and societal challenges will help advance robot-assisted surgery for PCa, benefiting patients.

### Supplementary Information

**Additional file 1: Fig. S1** Characteristics of robotic surgical system. **Table S1** Each generation of da Vinci robot-assisted surgical system and its characteristics.

## Data Availability

The datasets are available from the corresponding author on reasonable request.

## Abbreviations

AI: Artificial intelligence
BCR: Biochemical recurrence
3D: Three-dimensional
FDA: Food and Drug Administration
MP: Multi-port
RARP: Robot-assisted radical prostatectomy
PCa: Prostate cancer
PSM: Positive surgical margins
SP: Single-port
