# Supplementary material for: Intelligent medicine in focus: the 5 stages of evolution in robot-assisted surgery for prostate cancer in the past 20 years and future implications
Source: Mil Med Res. 2024 Aug 21;11:58. doi: 10.1186/s40779-024-00566-z (PMC11337898; doi:10.1186/s40779-024-00566-z)
Supplement: Supplementary file 1 — Additional file 1: Fig. S1 Characteristics of robotic surgical system. Table S1 Each generation of da Vinci robot-assisted surgical system and its characteristics. [file 40779_2024_566_MOESM1_ESM.pdf]

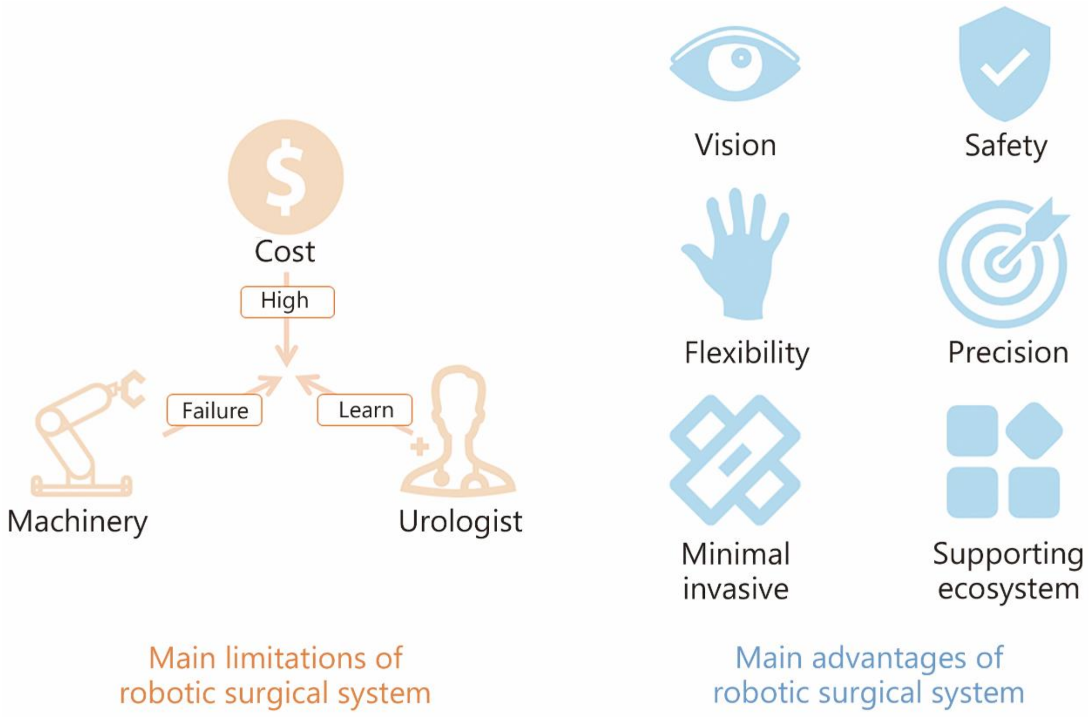

Fig. S1 Characteristics of robotic surgical system. Six significant advantages and three relatively weak aspects of the robotic surgical system. The blue outer circle in the figure showed the advantages of the robotic surgical system, while the orange-red contents in the inner circle showed the weak aspects

Table S1 Each generation of da Vinci robot-assisted surgical system and its characteristics

Time	Generation	Model	Characteristic
1996	1st	da Vinci	The first generation of da Vinci robotic system
2005	2nd	da Vinci S	The robotic arm had a larger range of motion, allowing doctors to observe multiple images without leaving the control console
2009	3rd	da Vinci Si	Added functions such as dual consoles, analog controllers, and intraoperative fluorescence development technology
2014	4th	da Vinci Xi	1) There have been qualitative improvements in flexibility, accuracy, imaging clarity, and other aspects<br>2) This version is the flagship version and the most versatile and powerful system in the fourth generation, which can be used for various surgeries
2017	4th	da Vinci X	1) It is a comprehensive version of da Vinci Xi and da Vinci Si, maintaining the basic functions of da Vinci Xi<br>2) The price and functions had decreased compared with da Vinci Xi, and the price is more affordable
2018	4th	da Vinci SP	Used for narrow abdominal surgery and oral, ear, and nasal surgery
2019	4th	da Vinci Endoscope Plus	Enhanced clarity and color accuracy
